# Dysregulation of autophagy during photoaging reduce oxidative stress and inflammatory damage caused by UV

**DOI:** 10.3389/fphar.2025.1562845

**Published:** 2025-05-12

**Authors:** Zhongsong Zhang, Run Tan, Zuanyu Xiong, Yanyan Feng, Long Chen

**Affiliations:** ^1^ School of Basic Medical Sciences, Chengdu Medical College, Chengdu, China; ^2^ Department of Dermatology, Chengdu Second People‘s Hospital, Chengdu, Sichuan Province, China; ^3^ School of Clinical Medicine, Chengdu Medical College, Chengdu, China; ^4^ Department of Medical Aesthetics, Nanbu People‘s Hospital, Nanchong, China

**Keywords:** photoaging, autophagy, UV, ROS, skin aging

## Abstract

Photoaging, the premature aging of skin due to chronic ultraviolet (UV) exposure, is a growing concern in dermatology and cosmetic science. While UV radiation is known to induce DNA damage, oxidative stress, and inflammation in skin cells, recent research unveils a promising countermeasure: autophagy. This review explores the intricate relationship between autophagy and photoaging, highlighting how this cellular recycling process can mitigate UV-induced damage. We begin by examining the differential impacts of UVA and UVB radiation on skin cells and the role of oxidative stress in accelerating photoaging. Next, we delve into the molecular mechanisms of autophagy, including its various forms and regulatory pathways. Central to this review is the discussion of autophagy’s protective functions, such as the clearance of damaged organelles and proteins, and its role in maintaining genomic integrity. Furthermore, we address the current challenges in harnessing autophagy for therapeutic purposes, including the need for selective autophagy inducers and a deeper understanding of its context-dependent effects. By synthesizing recent advancements and proposing future research directions, this review underscores the potential of autophagy modulation as a novel strategy to prevent and treat photoaging. This comprehensive analysis aims to inspire further investigation into autophagy-based interventions, offering new hope for preserving skin health in the face of environmental stressors.

## Graphical Abstract

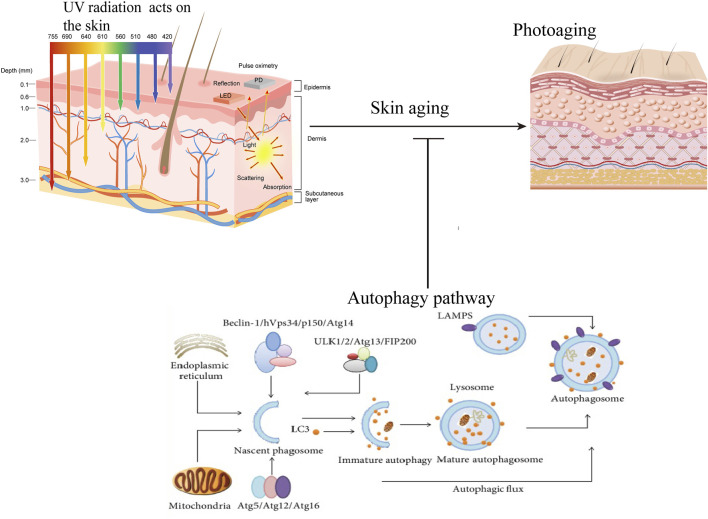

## 1 Introduction

Photoaging, the premature aging of the skin due to prolonged exposure to ultraviolet (UV) radiation, represents a major concern in dermatology and cosmetic science (Havas et al., 2022; Jin et al., 2022). It is estimated that approximately 80% of visible skin aging signs are caused by UV exposure, with photoaging being more prevalent in regions with high UV radiation exposure, such as tropical and subtropical areas (Yaar and Gilchrest, 2007; Krutmann et al., 2017). This condition arises from morphological and functional changes in the skin, influenced by a complex interplay of factors including genetic susceptibility, environmental stressors, and lifestyle choices (Ryu et al., 2022; Xu et al., 2022b; Yoon et al., 2022). Beyond its cosmetic implications, photoaging impacts health, psychological wellbeing, and socio-economic status, as visible aging signs can diminish self-esteem and affect social interactions (Shivakumar and Jafferany, 2020). Understanding its mechanisms is thus critical for both scientific inquiry and practical applications. A key cellular process implicated in this context is autophagy, a tightly regulated mechanism that degrades and recycles damaged organelles and misfolded proteins to maintain cellular homeostasis (Tang et al., 2021; Song et al., 2024). Autophagy plays an essential role in responding to stressors like oxidative damage, which is a hallmark of UV-induced skin aging (Machala et al., 2019; Ocansey et al., 2024). The relationship between photoaging and autophagy is particularly significant. For instance, UV exposure triggers oxidative stress that disrupts autophagic processes, leading to exacerbated cellular damage and perpetuating a cycle that amplifies photoaging effects (Popp et al., 2018; Kikuchi et al., 2020). Conversely, enhancing autophagic activity has emerged as a promising therapeutic strategy to mitigate these effects, underscoring its protective role in preserving skin integrity (Arensman and Eng, 2018).

Despite the individual attention given to photoaging and autophagy, their intersection remains underexplored, leaving a notable gap in the literature (Arensman and Eng, 2018; Arzalluz-Luque and Conesa, 2018). Current treatments for photoaging—such as topical antioxidants, retinoids, sunscreens, and procedures like chemical peels and laser therapies—focus primarily on reducing UV damage (Chan et al., 2024). However, emerging research suggests that targeting autophagy could offer novel avenues for intervention. For instance, studies using autophagy-enhancing compounds like rapamycin and metformin have shown encouraging results, improving skin health and reducing aging signs (Bharath et al., 2020; Ma J. et al., 2022; Ikutama et al., 2023). This highlights the potential of autophagy as a cornerstone for innovative anti-aging strategies.

This review aims to provide a comprehensive overview of the interplay between autophagy and photoaging. By examining the underlying mechanisms, synthesizing current research, and identifying knowledge gaps, we seek to lay the groundwork for future studies that could lead to effective therapies. Ultimately, our goal is to explore how autophagy’s protective potential can be harnessed to counteract the detrimental effects of photoaging, offering new possibilities for maintaining skin health in the face of environmental challenges.

## 2 Molecular mechanisms of photoaging

Photoaging differs from intrinsic aging, as it is mainly caused by UV-induced molecular damage that impacts cellular components such as proteins, lipids, and DNA (Widmer et al., 2006; Seo et al., 2009). The mechanisms of photoaging are characterized by intricate molecular pathways, which involve the production of reactive oxygen species (ROS), which, along with the activation of specific signaling cascades (Fan et al., 2024; Lu et al., 2024), and the breakdown of the extracellular matrix (Tang Z. et al., 2024), play a central role in the process. Here, we focus on the detailed molecular mechanisms by which UVA and UVB radiation, particularly through ROS generation, contribute to skin aging.

### 2.1 The impact of UV radiation on skin

Under normal conditions, UV radiation is classified into three types based on wavelength by the WHO: UVC (100–280 nm), UVB (280–315 nm), and UVA (315–400 nm) (Al-Sadek and Yusuf, 2024). However, UVC radiation is largely absorbed by the Earth’s ozone layer and does not reach the surface under natural conditions. Therefore, this discussion focuses on UVA and UVB, which are the primary contributors to photoaging through distinct mechanisms determined by their wavelength-related energy properties and penetration depths into the skin. UVA and UVB differ in their energy levels and penetration depths, leading to distinct mechanisms of skin damage. Shorter wavelengths, such as those of UVB, carry higher energy, while longer wavelengths, like UVA, penetrate deeper into the skin but with lower energy (Gęgotek and Skrzydlewska, 2022; Salminen et al., 2022; Tang X. et al., 2024) (Table 1). UVA constitutes about 95% of the UV radiation that reaches the Earth’s surface (Hargreaves et al., 2007; Narayanapillai et al., 2012), it penetrates deeper into the dermis where collagen, elastin, and fibroblasts are located (Xia et al., 2024; Yuan et al., 2024). Although it has lower energy than UVB, UVA induces significant damage over time by generating ROS, which cause oxidative damage to cellular components such as proteins, lipids, and DNA (Bernerd et al., 2022; Negre-Salvayre and Salvayre, 2022). This oxidative damage compromises the structural integrity and function of dermal cells, leading to visible signs of aging such as loss of skin elasticity, wrinkle formation, and collagen degradation over time. In contrast to UVA, UVB accounts for only about 5% of UV radiation but possesses higher energy, primarily affecting the epidermis (Calzari et al., 2023). The higher energy of UVB directly damages DNA by inducing cyclobutane pyrimidine dimers (CPDs) and pyrimidine (6-4) photoproducts, leading to mutations that can trigger carcinogenesis and inflammatory responses (Wang Z. et al., 2022; Oulee et al., 2023). Additionally, UVB exposure stimulates keratinocyte apoptosis and activates inflammatory cytokines (Yano et al., 2003; Glady et al., 2018; Min et al., 2022), further accelerating skin damage. UVB radiation is primarily responsible for sunburn. Chronic exposure to UVB can also lead to thickening of the epidermis, pigment changes, and premature aging (Ichihashi and Ando, 2014). Understanding the distinct mechanisms of UVA and UVB radiation—including their energy levels, penetration depths, and types of molecular damage—is crucial for comprehending how UV radiation leads to molecular damage and ultimately results in photoaging.

**TABLE 1:** Comparative summary of UVA and UVB: physicochemical parameters, molecular targets, biochemical pathways, and pathological outcomes.

S. No.	Factor	UVA	UVB	References
1	Wavelength	320–400 nm	280–320 nm	Diffey (2015), Camillo et al. (2022)
2	Penetration depth	Deep (dermis)	Shallow (epidermis)	Fernández-Carro and Ciriza (2025)
3	Photon energy	Lower energy photons (longer wavelengths)	Higher energy photons (shorter wavelengths)	Neale et al. (2023)
4	Primary molecular damage	ROS generation, oxidative damage to DNA, proteins, and lipids	Direct DNA damage (CPDs), ROS generation	Harwansh and Deshmukh (2023)
5	Biochemical pathways	Oxidative stress; ECM degradation; activation of MMPs	DNA repair (CPD excision), inflammation (cytokine release)	Zhao et al. (2025)
6	Pathological outcomes	Dermal damage, collagen breakdown, wrinkles	Epidermal inflammation, DNA mutations, sunburn	Kunisada et al. (2017)

Photon energy: refers to the relationship between wavelength and energy; shorter wavelengths (e.g., UVB) have higher energy photons capable of directly damaging DNA, while longer wavelengths (e.g., UVA) have lower energy photons that primarily induce oxidative stress through ROS, generation.

Thus, UVA predominantly contributes to deep dermal damage and oxidative stress by generating ROS that degrade structural proteins like collagen and elastin. In contrast, UVB primarily causes direct DNA damage in the epidermis through the formation of CPDs and induces epidermal inflammation. Both types of radiation act synergistically to accelerate skin aging, a process encompassing both intrinsic aging—caused by genetic and physiological factors—and extrinsic aging, driven by environmental influences such as chronic UV exposure.

### 2.2 ROS generation and damage

One of the key contributors to photoaging is the UV-induced generation of ROS, which play a pivotal role in initiating and propagating molecular damage in skin cells (Son et al., 2024; Yu Z.-W. et al., 2024). Both UVA and UVB radiation can lead to the formation of ROS, but the mechanisms and subsequent cellular effects differ between the two types of UV radiation (Svobodova et al., 2006; Manosalva et al., 2024). While UVB directly damages DNA by forming cyclobutane pyrimidine dimers (CPDs), UVA does not cause such direct DNA damage. Instead, UVA penetrates deeply into the skin and interacts with endogenous chromophores (e.g., flavins, porphyrins) to induce oxidative stress (Brem et al., 2017; Yagura et al., 2017; Bernerd et al., 2022). This interaction leads to the generation of ROS, including superoxide anions (O_2_^−^), hydroxyl radicals (⋅OH), and hydrogen peroxide (H_2_O_2_) (Zou et al., 2017; Brown et al., 2021). These ROS contribute to widespread oxidative damage by initiating lipid peroxidation, protein oxidation, and indirect DNA damage. For example, ROS can oxidize guanine bases in DNA, forming 8-oxo-deoxyguanosine (8-oxo-dG), a marker of oxidative DNA damage associated with mutagenesis (Bacqueville et al., 2021; Wang et al., 2024b).

VB radiation primarily damages DNA directly by forming CPDs when UVB photons are absorbed by DNA bases, leading to covalent bonds between adjacent pyrimidine bases. Beyond this direct damage, UVB also induces the generation of ROS by activating enzymatic pathways such as NADPH oxidases in keratinocytes. ROS are highly reactive molecules that disrupt cellular homeostasis, leading to oxidative stress—a state where the cellular antioxidant defenses are overwhelmed, resulting in damage to DNA, proteins, and lipids (Oulee et al., 2023; Al-Sadek and Yusuf, 2024; Pellacani et al., 2024). Specifically, UVB-induced ROS cause additional DNA damage beyond the initial CPDs, such as strand breaks and base oxidations, through a process known as secondary oxidative stress (Tuteja et al., 2009). This secondary oxidative stress arises when ROS generated by UVB exposure exacerbate initial cellular damage, contrasting with primary oxidative stress, which refers to the immediate oxidative damage caused directly by ROS upon their generation. Table 2 summarizes the mechanisms and consequences of ROS damage on DNA, proteins, and lipids.

**TABLE 2:** DNA, protein and lipid damage induced by ROS.

S. No.	Type of damage	Mechanism	Consequences	Potential interventions	References
1	DNA damage	UVA induces oxidative stress forming 8-oxo-dG, causing replication errors. UVB causes direct structural alterations (CPDs) in DNA.	Mutations - increased risk of photoaging and skin cancer	Antioxidants (vitamin C, vitamin E), UV-blocking agents, DNA repair enhancers (e.g., photolyases).	Zhou et al. (2012), Oulee et al. (2023), Al-Sadek and Yusuf (2024), Pellacani et al. (2024)
2	Protein damage	ROS upregulate MMPs (e.g., MMP-1 and MMP-3), leading to the breakdown of collagen and elastin.	Wrinkle formation Loss of skin firmness.	MMP inhibitors, retinoids, peptide-based therapies.	Lee et al. (2022), Kim et al. (2024a)
3	Lipid peroxidation	ROS cause peroxidation of cellular membrane lipids, altering permeability and disrupting signaling pathways.	Cellular dysfunction Cytotoxic effects and inflammation in photoaging.	Antioxidants, fatty acid supplementation, anti-inflammatory agents.	Briganti and Picardo (2003), Dissemond et al. (2003), Damiani et al. (2006)

In conclusion, UVA and UVB accelerate photoaging through complementary mechanisms: UVA penetrates the dermis to generate ROS, causing oxidative stress and structural protein degradation, while UVB affects the epidermis by directly damaging DNA, triggering inflammation and hyperproliferation, together contributing to skin aging. Future research should further elucidate the molecular pathways linking UVA and UVB exposure to specific photoaging outcomes, enabling more targeted prevention and intervention strategies (Kim et al., 2022).

### 2.3 Signaling pathways associated with photoaging

Photoaging results from signaling pathways activated by UV radiation, which cause cellular damage, inflammation, and aging (Li et al., 2024c; Wang K. et al., 2024). Two of the most well-characterized pathways are the Mitogen-Activated Protein Kinase (MAPK) and Nuclear Factor kappa-light-chain-enhancer of activated B cells (NF-κB) pathways (Ge et al., 2024; Zhang et al., 2024). These pathways regulate the expression of matrix metalloproteinases (MMPs), enzymes that degrade collagen and elastic fibers, thereby contributing to the structural damage characteristic of photoaged skin (Han et al., 2022). The breakdown of these extracellular matrix components weakens the skin’s structural integrity and promotes wrinkle formation, which are hallmark features of photoaging (Hajialiasgary Najafabadi et al., 2024).

#### 2.3.1 MAPK signaling pathway

The MAPK signaling pathway plays a pivotal role in mediating cellular responses to various stressors, including UV radiation (Bosch et al., 2015). UV exposure activates several MAPK family members—ERK (Extracellular Signal-Regulated Kinase), JNK (c-Jun N-terminal Kinase), and p38 MAPK—through a series of upstream signaling events (Madson and Hansen, 2007; López-Camarillo et al., 2012). UV radiation induces DNA damage, oxidative stress, and the activation of cell surface receptors such as epidermal growth factor receptor (EGFR). These receptors trigger a cascade of phosphorylation events mediated by small GTPases like Ras and Rac, leading to the sequential activation of MAPK kinase kinases (MAP3Ks), MAPK kinases (MAP2Ks), and ultimately MAPKs. Each of these kinases has distinct roles in the context of photoaging. ERK is primarily involved in cell proliferation and survival (Cagnol and Chambard, 2010; Sun et al., 2015). UV-induced activation of ERK promotes cell cycle progression and proliferation (Djavaheri-Mergny and Dubertret, 2001). However, prolonged or chronic activation, often resulting from repeated UV exposure, can lead to cellular senescence—a hallmark of aging characterized by irreversible cell cycle arrest and the secretion of pro-inflammatory factors, known as the senescence-associated secretory phenotype (SASP) (Victorelli et al., 2023; Ouvrier et al., 2024). JNK is activated by stress signals, including UV radiation. Once activated, JNK promotes the expression of pro-inflammatory cytokines and mediates apoptosis in severely damaged cells (Chen et al., 2018b; Zhang C. et al., 2022). This pathway contributes to the inflammatory response associated with photoaging and is linked to the induction of senescence in keratinocytes (Piao et al., 2015; Wu et al., 2016). p38 MAPK is particularly responsive to UV-induced stress and plays a critical role in regulating inflammatory responses (Jinlian et al., 2007). It activates transcription factors that induce the expression of MMPs (matrix metalloproteinases) and other inflammatory mediators (Mavrogonatou et al., 2018), facilitating collagen degradation and the subsequent loss of skin elasticity. Chronic activation of p38 is associated with sustained inflammatory states, exacerbating skin aging (Lee et al., 2009; Li H. et al., 2024). The proposed mechanism involves MAPK activation leading to a cascade of events that impact cellular responses in photoaging. Specifically, MAPK pathways contribute to oxidative stress, inflammation, and matrix degradation (Xu et al., 2022a). ERK promotes cell survival and proliferation, while prolonged activation may lead to senescence (Byun et al., 2023). JNK contributes to inflammation and apoptosis, and p38 MAPK regulates inflammation and collagen degradation (Ma et al., 2011). This coordinated activation of pathways under UV stress ultimately accelerates skin aging.

Thus, the MAPK signaling pathway serves as a crucial mediator of the cellular response to UV radiation, orchestrating a balance between proliferation, survival, inflammation, and cellular senescence (Yan et al., 2023; Yang T. et al., 2024). Dysregulation of this pathway can lead to enhanced photoaging and skin damage.

#### 2.3.2 NF-κB signaling pathway

The interaction between NF-κB and MAPK pathways highlights the complex signaling involved in UV exposure, linking inflammation, cellular damage, and aging. The NF-κB signaling pathway plays a critical role in the inflammatory response to UV radiation (Bang et al., 2021; Han et al., 2022). Upon UV exposure, NF-κB is activated through phosphorylation events involving IκB kinase (IKK), leading to the degradation of IκB proteins and translocation of NF-κB into the nucleus (Tsuchiya et al., 2010; Bang et al., 2021). Once there, NF-κB promotes the transcription of inflammatory cytokines such as TNF-α, IL-1, and IL-6, which amplify inflammation and attract immune cells like neutrophils and macrophages to the damaged site (Mussbacher et al., 2023; Nam, 2006; Wang Z. et al., 2024). This recruitment of immune cells and the subsequent release of pro-inflammatory cytokines and ROS play a central role in perpetuating inflammation and oxidative stress. This inflammatory environment exacerbates the oxidative stress and cellular damage initiated by UV radiation by promoting the release of additional ROS and nitrogen species from recruited immune cells (Kim et al., 2022). The elevated ROS levels overwhelm the cellular antioxidant defenses, creating a feedback loop where oxidative stress amplifies inflammation, perpetuating cellular damage and structural degradation. Chronic activation of NF-κB contributes significantly to extracellular matrix breakdown. Chronic activation of NF-κB, through sustained inflammatory responses, contributes to the breakdown of the extracellular matrix, as it enhances the transcription of MMP (Yuan et al., 2022). While sustained expression of inflammatory cytokines leads to increased transcription of MMPs, MMPs are synthesized as inactive zymogens that require post-translational activation (Tanaka et al., 2010; Fu et al., 2024). This activation process involves proteolytic cleavage by enzymes such as plasmin or other active MMPs, as well as oxidative modifications induced by ROS, which enhance the proteolytic activity of MMPs (Tanaka et al., 2010; Liu S. et al., 2022). Once activated, MMPs degrade collagen, elastin, and other extracellular matrix components, weakening the skin’s structural integrity and contributing to wrinkle formation and the development of solar elastosis (Heng, 2013). Thus, NF-κB activation plays a critical role in both initiating and amplifying the inflammatory cascade that accelerates photoaging by promoting matrix degradation and enhancing ROS production. This highlights that both increased MMP transcription and their subsequent activation are critical steps in the progression of photoaging.

#### 2.3.3 UV radiation-driven signaling pathways contributing to photoaging

**FIGURE 1:**
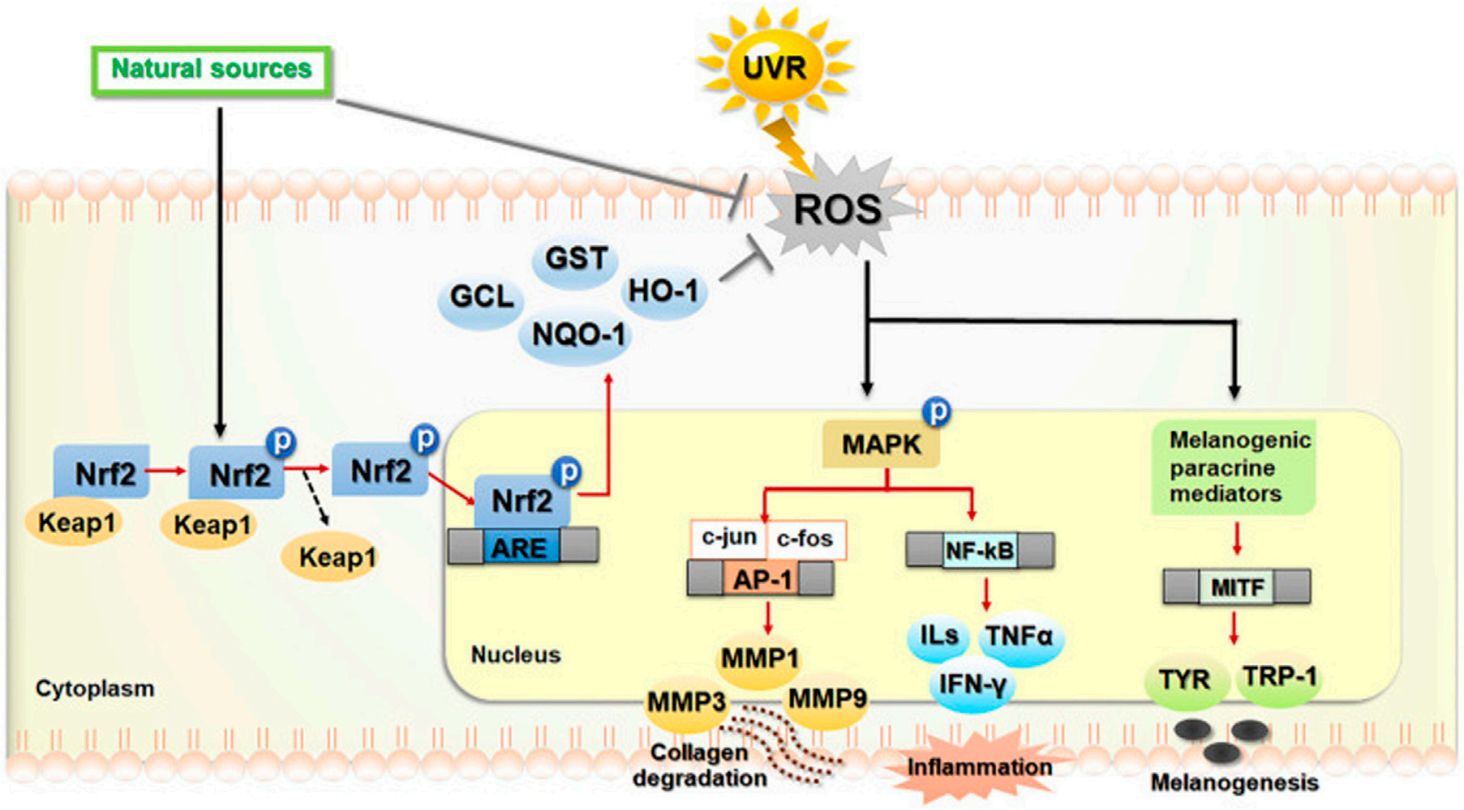
UV radiation-driven signaling pathways contributing to photoaging. The extended content further elaborates on the roles of specific signaling pathways in photoaging. It maintains a strong academic tone by detailing how each pathway (Nrf2, MAPK, NF-κB) contributes to different aspects of skin damage and aging. This expanded explanation provides a clear and coherent description of the complex molecular events involved, ensuring that readers can follow the intricate relationships between ROS, cellular signaling, and the eventual skin aging process caused by UV exposure. Abbreviations: UVR: Ultraviolet Radiation; ROS: Reactive Oxygen Species; Nrf2: Nuclear factor erythroid 2-related factor 2; Keap1: Kelch-like ECH-associated protein 1; GCL: Glutamate-cysteine ligase; GST: Glutathione S-transferase; HO-1: Heme oxygenase 1; NQO-1: NAD(P)H quinone dehydrogenase 1; MAPK: Mitogen-activated protein kinase; ERK: Extracellular signal-regulated kinase; JNK: c-Jun N-terminal kinase; p38: p38 mitogen-activated protein kinase; AP-1: Activator protein 1; NF-κB: Nuclear factor kappa-light-chain-enhancer of activated B cells; MMPs: Matrix metalloproteinases; MMP1: Matrix metalloproteinase 1; MMP3: Matrix metalloproteinase 3; MMP9: Matrix metalloproteinase 9; ILs: Interleukins; TNF-α: Tumor necrosis factor alpha; IFN-γ: Interferon gamma; MITF: Microphthalmia-associated transcription factor; TYR: Tyrosinase; TRP-1: Tyrosinase-related protein 1. Reproduced from Chaiprasongsuk and Panich (2022). Role of Phytochemicals in Skin Photoprotection via Regulation of Nrf2. Frontiers in pharmacology, 13, 823881. Copyright ^©^ 2022 by (Chaiprasongsuk and Panich, 2022).

Photoaging is a complex process influenced by various signaling pathways activated in response to UVR. These pathways can either protect against or contribute to cellular damage, inflammation, and the eventual aging of skin tissue (De Magis et al., 2023) (Figure 1). UVR generates ROS, which activate several critical cellular pathways that modulate the skin’s response to damage. One of the key protective pathways is the Nrf2 pathway (Kahremany et al., 2022). In response to oxidative stress induced by ROS, Nrf2 dissociates from the Keap1 complex, translocases into the nucleus, and triggers the transcription of various antioxidant enzymes such as GCL, GST, HO-1, and NQO-1 (Kahremany et al., 2022; Robinson et al., 2022; Cai et al., 2023). These enzymes help mitigate ROS-induced cellular damage and play an essential role in maintaining cellular homeostasis.

Conversely, UVR also activates the MAPK signaling pathway, which involves three major kinases: ERK, JNK, and p38 (Xu et al., 2022c). The activation of these kinases leads to the downstream activation of key transcription factors, including AP-1 and NF-Κb (Kim K. et al., 2024). These factors regulate inflammatory responses and contribute to the degradation of the extracellular matrix, a crucial event in the aging process of skin cells. AP-1 activation increases the expression of MMPs, such as MMP1, MMP3, and MMP9, which degrade collagen and elastin, leading to skin’s loss of structural integrity and the formation of wrinkles (Liu Y. et al., 2022; Zhang et al., 2024). Additionally, NF-κB activation further exacerbates the inflammatory environment by upregulating pro-inflammatory cytokines like interleukins (ILs), TNF-α, and IFN-γ (Qu et al., 2022; Yuksel Egrilmez et al., 2022). This cascade amplifies cellular and tissue damage, contributing to the breakdown of skin architecture and accelerating photoaging. Furthermore, ROS can stimulate the melanogenic pathway, leading to increased activity or expression of mediators such as MITF, tyrosinase, TYR, and TRP-1, which regulate pigmentation changes in the skin (Charachit et al., 2022). This pathway, in combination with the other signaling events, plays a pivotal role in the appearance of age spots and uneven pigmentation, which are hallmarks of photoaging (Li et al., 2024d).

In summary, UV radiation-induced ROS activates the protective Nrf2 pathway, reducing damage; MAPK pathway promotes inflammation; The NF-κB pathway leads to inflammation and matrix degradation; and the melanin production pathway, which triggers changes in pigmentation. The balance of these pathways determines the degree of photoaging, including wrinkle formation, decreased skin elasticity, and pigment changes, and provides potential targets for prevention or treatment strategies.

#### 2.3.4 Collagen degradation and elastic fiber damage in photoaging

Photoaging is characterized by the breakdown of collagen and damage to elastic fibers, primarily due to the activity of enzymes such as MMPs (Kong et al., 2023; Jariwala et al., 2024). UV radiation triggers a series of events that lead to the upregulation and activation of these enzymes. Specifically, UV exposure increases the expression of MMPs, including MMP-1 (collagenase), MMP-3 (stromelysin), and MMP-9 (gelatinase), which degrade collagen and other extracellular matrix components (Lee et al., 2023; Oh S. et al., 2023; Feng C. et al., 2024). These enzymes degrade collagen and other extracellular matrix components, resulting in structural changes in the skin (Jariwala et al., 2024). For example, MMP-1 specifically targets type I and III collagen, which are the major components of the dermal matrix, leading to the typical loss of skin firmness and elasticity seen in photoaged skin (Robichaud et al., 2011). In addition to collagen, MMPs also play a role in the breakdown of elastic fibers (Xiong et al., 2008; Ribeiro-Silva et al., 2021). UV radiation promotes the expression of elastases, enzymes that specifically degrade elastin. The destruction of elastic fibers leads to a reduction in skin turgor and resilience, contributing to the formation of fine lines and sagging skin (Sadick et al., 2023; Jariwala et al., 2024).

The activity of MMPs is influenced by signaling pathways activated by UV exposure. For example, the NF-κB pathway can induce the expression of inflammatory cytokines, which subsequently activate MMPs, while the p38 MAPK pathway plays a role in the activation of MMPs through post-translational modifications. These signaling pathways play an integral role in amplifying the inflammatory response and contributing to the chronic activation of MMPs. The activation of MMPs is closely linked to the signaling pathways activated by UV exposure (Yuksel Egrilmez et al., 2022; Oh J. H. et al., 2023). These pathways are involved in the inflammatory response, which can indirectly influence the activation of MMPs and contribute to the degradation of collagen and elastic fibers in photoaged skin. For example, the NF-κB pathway stimulates the expression of MMPs in response to inflammatory cytokines, while the p38 MAPK pathway enhances MMP transcription as part of the cellular stress response (Li et al., 2022; Zhang Y. et al., 2022; Jeon et al., 2024). This coordinated response ultimately leads to a significant alteration of the extracellular matrix, resulting in photoaging.

In conclusion, the signaling pathways triggered by UV radiation, especially the MAPK and NF-κB pathways, are crucial in mediating the cellular response to UV-induced damage (Ge et al., 2024). These pathways orchestrate a feedback loop between inflammation, oxidative stress, and MMP activation, driving the degradation of collagen and elastic fibers (Kim et al., 2014; Liu et al., 2019). This cascade results in the characteristic features of photoaged skin (Li L. et al., 2019; Choi et al., 2020) (Table 3). Gaining a deeper understanding of these mechanisms offers valuable insights into potential therapeutic approaches to counteract the effects of photoaging and maintain skin health.

**TABLE 3:** Signal pathways triggered by UV radiation and their role in photoaging.

S. No.	Signaling pathways	Activation mechanism	Function	Association with photoaging	References
1	MAPK signaling pathway	UV radiation activates the ERK, JNK, and p38 MAPK pathways, leading to phosphorylation and activation of downstream targets.	Promotes cell proliferation and survival, inflammatory cytokine expression, and apoptosis. Regulates the inflammatory response.	Promoting cellular damage, inflammation and aging processes.	Jinlian et al. (2007), Cagnol and Chambard (2010), Sun et al. (2015), Nisar et al. (2022), Qu et al. (2022)
2	NF-κB signaling pathway	UV radiation triggered phosphorylation, promoted the translocation of nf- κ B into the nucleus.	Regulated the expression of inflammatory cytokines (such as tnf- α, IL-1, IL-6).	Chronic activation leads to MMP upregulation, accelerating collagen and elastic fiber degradation.	Nam (2006), Tanaka et al. (2010), Liu et al. (2022a), Chen et al. (2023b), Zhao et al. (2023), Wang et al. (2024f)
3	Collagen degradation and elastic fiber damage	UV radiation induces the activation of MMPs and elastase, which then degrade collagen and elastic fibers.	MMP-1 degrades type I and III collagen; elastase degrades elastin, causing loss of skin elasticity and firmness.	Leads to structural changes such as fine lines, wrinkles, and skin sagging.	Robichaud et al. (2011), Ribeiro-Silva et al. (2021), Sadick et al. (2023), Jariwala et al. (2024)

This table summarizes the activation mechanism and function of MAPK, and NF-κB, signaling pathways under UV, radiation and their importance in photoaging.

### 2.4 Epigenetics and photoaging

Epigenetics refers to mechanisms that alter gene expression through environmental factors or lifestyle choices without modifying the DNA sequence (He et al., 2022; Zand et al., 2024). Recently, interest in epigenetics has surged due to its role in various diseases and its influence on cellular responses to environmental stimuli, such as UV radiation, particularly in skin aging (Yuksel, 2020; Barnes et al., 2024). Research indicates that epigenetic modifications significantly affect how cells respond to UV radiation and contribute to photoaging progression (Lee Y. et al., 2021; Kim et al., 2023; Lin et al., 2024a). Two key mechanisms of epigenetic regulation, DNA methylation and histone modification, are particularly relevant in this context (Lee Y. et al., 2021; Zand et al., 2024). UV radiation can induce alterations in DNA methylation patterns, leading to changes in gene expression. For example, UV exposure has been shown to promote the hypermethylation of specific genes involved in the skin’s protective mechanisms and DNA repair processes (Crochemore et al., 2023; Johann To Berens et al., 2024). This hypermethylation silences essential genes, resulting in reduced expression of proteins critical for cellular repair and maintenance, thereby contributing to the aging process (de Oliveira et al., 2020). Such epigenetic modifications can increase the skin’s vulnerability to further UV damage, establishing a cycle of degradation (Qin H. et al., 2018). Similarly, UV radiation influences histone modifications, such as acetylation and methylation, which affect chromatin structure and gene accessibility (Xie L. et al., 2022; Wen et al., 2024). For instance, increased histone acetylation can enhance the expression of pro-inflammatory genes, while decreased acetylation may silence genes responsible for cell proliferation and repair (Lee Y. et al., 2021; Negre-Salvayre and Salvayre, 2022). Similarly, alterations in histone methylation can either activate or suppress gene expression, influencing processes critical to skin homeostasis (Negre-Salvayre and Salvayre, 2022).

Furthermore, epigenetic modifications interact with autophagy regulation, influencing photoaging (Durocher et al., 2019; Patra et al., 2019; Zha et al., 2019). For example, DNA methylation and histone modifications can affect the expression of autophagy-related genes (such as ATG genes) (Peixoto et al., 2019; Szustka et al., 2024), thereby regulating autophagic flux. Disruptions in autophagy due to these epigenetic changes can result in the accumulation of damaged proteins and organelles (Alves-Fernandes and Jasiulionis, 2019), which in turn exacerbates oxidative stress and cellular senescence. This interaction highlights the complexity of the mechanisms underlying photoaging and emphasizes the necessity for additional research to explore how these epigenetic alterations could be targeted to alleviate the effects of UV-induced skin aging (Jeremian et al., 2024; Lin et al., 2024a).

## 3 Molecular mechanisms of autophagy pathways

### 3.1 Classification of autophagy

**FIGURE 2:**
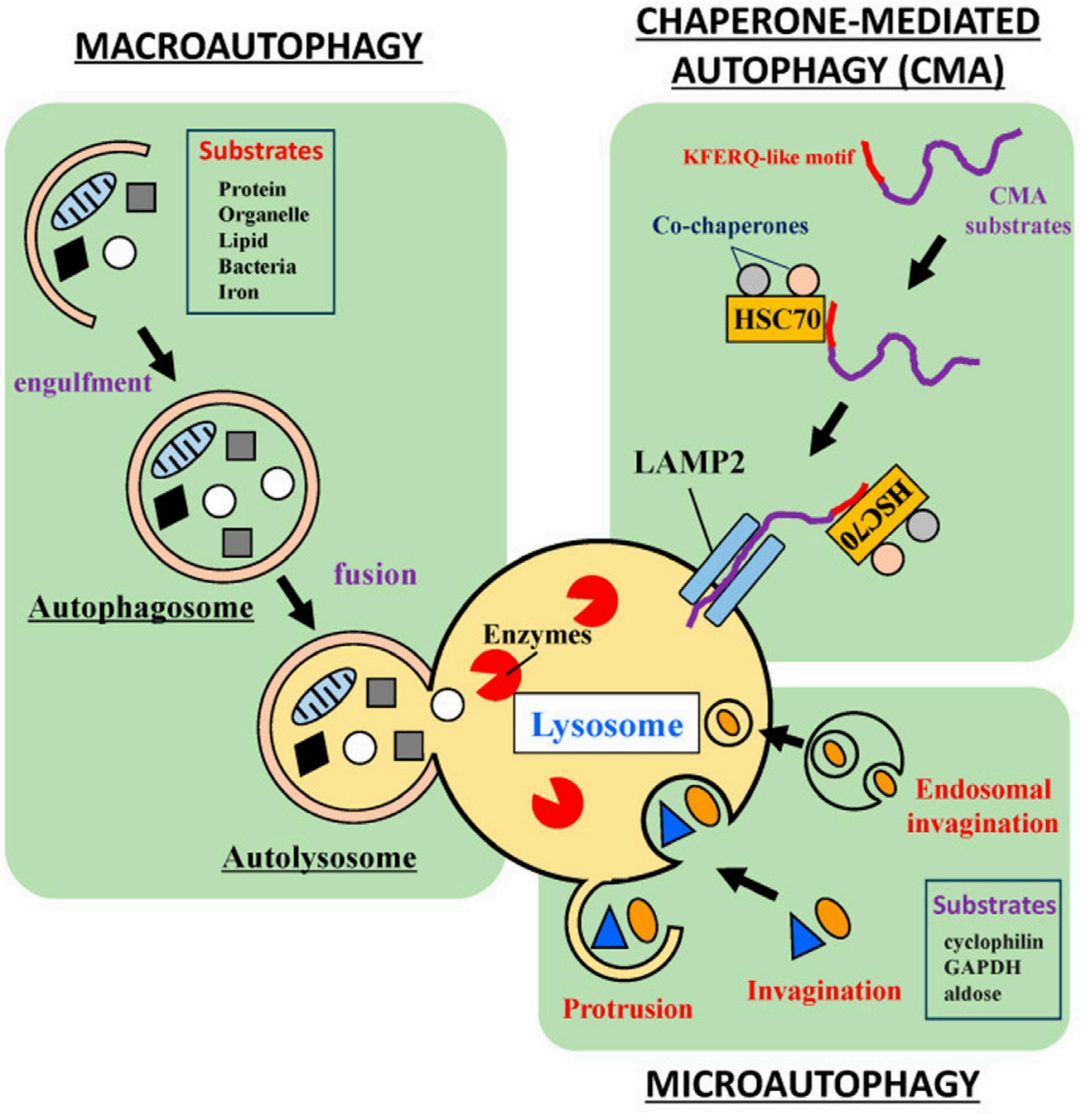
Classification of autophagy. Three main autophagy subtypes involved in cellular metabolism and balance, including macroautophagy, microautophagy, and a selective autophagy process: Chaperone-Mediated Autophagy (CMA). Reproduced from Watanabe et al. (2023). Roles of Stress Response in Autophagy Processes and Aging-Related Diseases. International journal of molecular sciences vol. 24, 18 13804. 7, Copyright ^©^ 2023 by (Yan et al., 2023).

Autophagy is a highly regulated cellular degradation process that maintains cellular homeostasis by removing damaged organelles, misfolded proteins, and other cellular debris (Chen et al., 2024; Kim J. et al., 2024). It can be broadly classified into three types: macroautophagy, microautophagy, and selective autophagy (Yan et al., 2023; Yamamoto and Matsui, 2024). Each type possesses unique characteristics and mechanisms, which are essential for the specific cellular contexts in which they function. Next, we will introduce these different autophagy types (Figure 2).

At present, macroautophagy, the most extensively studied form of autophagy, involves the formation of double-membrane structures called autophagosomes (Klionsky and Codogno, 2013; Feng Y. et al., 2024). Macroautophagy begins with the induction of autophagy, often triggered by cellular stressors such as nutrient deprivation or oxidative stress (Lyamzaev et al., 2018; Beccari et al., 2023). Oftentimes, the initiation of macroautophagy is regulated by various signaling pathways (Popelka and Klionsky, 2015), primarily the mechanistic target of rapamycin (mTOR) pathway (Pattingre et al., 2008; Ott et al., 2016). Once the process begins, the isolation membrane, referred to as the phagophore, elongates to enclose cytoplasmic components, ultimately completing the formation of an autophagosome (Egan et al., 2015; Wang et al., 2018). The autophagosome then fuses with a lysosome to form an autolysosome, where lysosomal hydrolases degrade the contents, and the resulting breakdown products are recycled back into the cytosol (Cao et al., 2021; Barnaba et al., 2023).

Microautophagy, unlike macroautophagy, involves the direct invagination of the lysosomal membrane to engulf cytoplasmic components (Pereira et al., 2012; Lemasters, 2014; Oku et al., 2017). This process occurs through the protrusion of lysosomal membrane invaginations, which directly internalize the cargo (Wang L. et al., 2023; Yamamoto and Matsui, 2024). While microautophagy is not as extensively studied as macroautophagy, it is thought to contribute to the breakdown of smaller proteins and organelles (Kuchitsu and Taguchi, 2024; Yamamoto and Matsui, 2024). Importantly, this type of autophagy is considered a constitutive process and may operate continuously to support the maintenance of cellular homeostasis.

Selective autophagy encompasses a variety of mechanisms that target specific cellular components for degradation, and one of the most common forms is chaperone-mediated autophagy (CMA) (Krause et al., 2023; Yan et al., 2023). This also includes the removal of damaged mitochondria (mitophagy), peroxisomes (pexophagy), and protein aggregates (aggrephagy) (Guan et al., 2022; Rubio-Tomás et al., 2023; Bajdzienko and Bremm, 2024). Pexophagy specifically targets damaged peroxisomes, which are organelles responsible for detoxifying ROS and metabolizing fatty acids (Dolese et al., 2022; Manandhar et al., 2024). In the context of photoaging, pexophagy helps prevent the accumulation of dysfunctional peroxisomes that can exacerbate oxidative stress and contribute to skin aging (Gallagher and Holzbaur, 2023). Aggrephagy, on the other hand, is responsible for the degradation of aggregated proteins, which may accumulate due to cellular stressors such as UV exposure (Li et al., 2023). The removal of these protein aggregates is critical for maintaining cellular integrity and preventing the detrimental effects of protein aggregation on skin cells (Rai et al., 2019). Different from macroautophagy and microautophagy, selective autophagy relies on specific receptors and cargo recognition mechanisms, ensuring that only designated targets are engulfed by the autophagic machinery (Mochida and Nakatogawa, 2022). For instance, mitophagy is facilitated by receptors such as PINK1 and Parkin, which are instrumental in marking dysfunctional mitochondria for degradation (Koentjoro et al., 2017; Narendra and Youle, 2024). Mitochondrial processes are particularly relevant in selective autophagy because mitochondria are not only key energy producers in the cell but also play a central role in maintaining cellular homeostasis (Yang et al., 2019; Feng Y. et al., 2024). In skin cells, mitochondria damaged by UV radiation may accumulate excessive ROS, exacerbating oxidative stress and leading to cellular damage, inflammation, and accelerated aging (Dunaway et al., 2018; Umar et al., 2019; Prasert et al., 2023). Mitophagy helps remove these damaged mitochondria, thereby maintaining cellular energy balance and preventing further functional decline during the photoaging process (Ding and Yin, 2012). In photoaging, impaired mitophagy can worsen oxidative damage in skin cells, promote collagen degradation, and damage elastic fibers, thus accelerating the appearance of aging signs (Panwar et al., 2018; Chen et al., 2020). Furthermore, an imbalance in mitophagy can contribute to various diseases, including neurodegenerative disorders, cancer, and aging, which are closely associated with the onset and progression of photoaging (Gaetano et al., 2021; Ghosh and Kumar, 2024). Therefore, mitophagy plays a crucial role in preserving mitochondrial integrity and maintaining overall cellular function, especially in skin cells exposed to UV-induced damage.

Macroautophagy is responsible for the large-scale degradation of cellular components through the formation of autophagosomes, whereas microautophagy operates via the direct inward folding of the lysosomal membrane. Selective autophagy specifically targets and eliminates particular cellular structures. A clear understanding of these distinctions is vital for unraveling the roles of autophagy in various physiological and pathological conditions. Recognizing these differences is essential for providing deeper insights into the involvement of autophagy in diverse biological processes.

### 3.2 Basic pathways of autophagy

**FIGURE 3:**
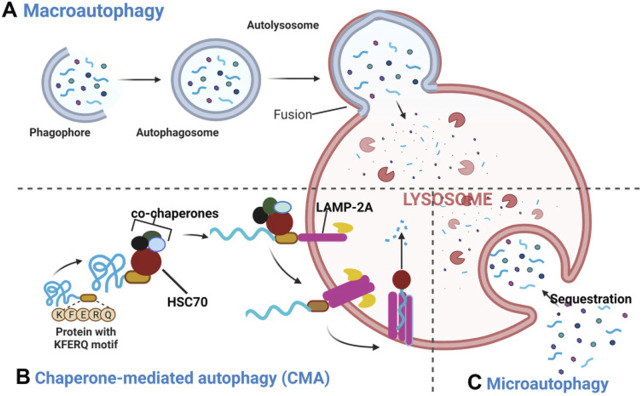
Macroautophagy, Microautophagy, and Selective Autophagy in the Autophagic Process. **(A)** Macroautophagy: during macroautophagy, cytosolic substrates, including proteins and organelles, are sequestered by the autophagosome. The fusion of the autophagosome with the lysosome to form the autolysosome is a crucial step for degradation, ensuring that the sequestered contents are efficiently processed. **(B)** Chaperone-Mediated Autophagy (a kind of Selective Autophagy): in CMA, proteins containing the pentapeptide KFERQ-like sequence are recognized by the Hsc70 chaperone. This chaperone binds to the target proteins and associates with the lysosomal membrane protein LAMP-2A, initiating its oligomerization. This event facilitates the translocation of the target protein into the lysosomal lumen, a process that is Hsc70-dependent. **(C)** Microautophagy: in microautophagy, lysosomes directly engulf cytosolic components through invagination or protrusion of the lysosomal membrane, without the prior formation of an autophagosome. This process allows for the direct sequestration of cellular material for degradation. Reproduced from Assaye M. A., Gizaw S. T. Chaperone-Mediated Autophagy and Its Implications for Neurodegeneration and Cancer. Int J Gen Med., Copyright ^©^ 2024 by (Assaye and Gizaw, 2022).

Autophagy involves five primary steps—induction, isolation, transport, degradation, and reutilization—that are essential to its three main types: macroautophagy, microautophagy, and selective autophagy (Table 4) (Wurzer et al., 2015; Bradley et al., 2022; Lim S. H. Y. et al., 2024; Majumder et al., 2024) These steps, illustrated in Figure 3, enable macroautophagy to degrade bulk cytoplasmic materials, microautophagy to engulf materials directly via the lysosomal membrane, and selective autophagy to target specific cellular components.

**TABLE 4:** Comparative table of autophagy processes.

S. No.	Step	Macroautophagy	Microautophagy	Selective autophagy	References
1	Induction	mTOR inhibition triggers the ULK1/ATG1 complex to initiate autophagy under stress (e.g., nutrient deprivation).	Constitutive process, occurs directly via lysosomal membrane invagination without complex signaling pathways.	Similar to macroautophagy, but with specific receptor ligand recognition (e.g., PINK1/Parkin in mitophagy).	Li et al. (2012), Li et al. (2018), Rui et al. (2015)
2	Isolation	Phagophore engulfs cargo, forms autophagosome. LC3-I mediates cargo sequestration and membrane elongation.	Lysosomal membrane directly invaginates and engulfs cytoplasm or organelles.	Cargo is selected based on receptor interactions (e.g., p62 binds ubiquitinated proteins for degradation).	Mizushima and Yoshimori (2007), Kumar et al. (2022), Yamamoto and Matsui (2024)
3	Transport	Autophagosome moves along microtubules, transported by dynein motors to fuse with lysosome.	No transport required; lysosome engulfs cargo directly.	Similar to macroautophagy, but involves organelle-specific transport mechanisms (e.9.mitochondrial transport).	Li et al. (2012), Djeddi et al. (2015), Magalhaes-Novais et al. (2022)
4	Degradation	Autophagosome fuses with lysosome; lysosomal hydrolases degrade contents inside autolysosome.	Lysosome engulfs cargo and immediately degrades it via hydrolases in the lysosomal lumen.	Similar to macroautophagy, but more selective for specific targets (e.g., mitochondria, protein aggregates).	Zhang et al. (2022b), Zhou et al. (2022), Vargas et al. (2023)
5	Reutilization	Degradation products (e.g., amino acids, fatty acids) are recycled back into the cytosol for energy/biosynthesis.	Breakdown products are reused, though at a smaller scale due to the nature of direct cytoplasmic invagination.	Degradation products are recycled depending on the specific organelle or structure degraded (e.g., mitophagy).	Kumariya et al. (2021), Zhou et al. (2022)

This table outlines the comparative processes of macroautophagy, microautophagy, and selective autophagy, highlighting their similarities and distinct mechanisms.

The process begins with the induction phase, where stressors like nutrient deprivation, oxidative stress, or hypoxia trigger autophagy These signals are recognized by receptors like mTOR (mechanistic target of rapamycin), which, when inhibited by stress signals, activates the autophagy machinery (Iannucci et al., 2021; Jarisarapurin et al., 2021). The isolation phase involves the formation of a membrane structure called the phagophore. This structure expands to surround cellular cargo, such as damaged organelles or protein aggregates, and sequesters them within the growing vesicle (Saetre et al., 2015; Schmitt et al., 2022; Shatz and Elazar, 2024). Following this, the transport phase occurs, where the autophagosome, now encapsulating the cargo, moves along microtubules toward the lysosome. This process is facilitated by motor proteins such as dynein and kinesin, which guide the autophagosome to the lysosome for fusion (Bozic et al., 2020). Once the autophagosome fuses with the lysosome, its contents are degraded within the autolysosome by hydrolytic enzymes. Finally, the reutilization phase involves the recycling of macromolecules such as amino acids, lipids, and sugars, which are released back into the cytosol for reuse in cellular metabolism (Feng et al., 2014; Cerda-Troncoso et al., 2020).

Recent studies have illuminated the intricate regulation of autophagy, showing how processes like macroautophagy, microautophagy, and selective autophagy are governed by signaling pathways such as mTOR, PI3K, and AMPK. These pathways coordinate the initiation, elongation, and maturation of autophagic vesicles in response to cellular stressors like nutrient deprivation, oxidative stress, and hypoxia (Beyaz et al., 2023; Chien et al., 2023; Kaur et al., 2023). For example, research by Inmaculada Navarro-Lérida et al. has illustrated how different autophagic pathways can be selectively activated by specific stimuli such as damage to organelles or the presence of protein aggregates, revealing the dynamic nature of autophagy in cellular adaptation and survival (Navarro-Lérida et al., 2022).

This deeper understanding of autophagic regulation enhances our grasp of cellular homeostasis and opens avenues for therapeutic interventions targeting autophagic dysfunction in diseases such as skin aging, photoaging, and neurodegeneration (Wang et al., 2019; Lim et al., 2020; Chu et al., 2023). By carefully examining these pathways and their roles in autophagy, researchers can gain deeper insights into the cellular processes underlying aging and disease, paving the way for future treatments.

### 3.3 Key regulatory molecules and signaling pathways

This section delves deeper into the molecular players and signaling pathways that regulate autophagy, providing a comprehensive understanding of their roles and interrelationships. Autophagy is a highly regulated process, controlled by various key molecules and signaling pathways that govern its initiation, progression, and completion (Selarka and Shravage, 2024; Wu et al., 2024; Yao et al., 2024). Critical regulators include the ATG gene family, the mTOR and AMPK pathways (Luo, 2014; Foerster et al., 2022), as well as recently discovered modulators like non-coding RNAs, which collectively ensure that autophagy occurs under the correct cellular conditions, such as nutrient deprivation or stress (Liu M. et al., 2024; Mirabdali et al., 2024). These regulators orchestrate the balance between cellular adaptation and homeostasis, playing an essential role in maintaining cellular function and survival (Rodríguez-Vargas et al., 2019; Yun et al., 2021) (Table 5).

**TABLE 5:** Key regulatory pathways in autophagy.

S. No.	Regulator	Function	Mechanism	Effect of autophagy	References
1	mTOR	Suppressor of autophagy	Phosphorylates and inhibits ULK1, blocking autophagosome formation under nutrient-rich conditions.	Inhibits (blocks autophagy initiation)	Rabanal-Ruiz et al. (2017), Kma and Baruah (2022)
2	AMPK	Activator of autophagy	Activates ULK1 and inhibits mTOR during energy stress by phosphorylating TSC2 and Raptor.	Activates (promotes autophagy under stress)	Allouch and Munusamy (2017), Anand et al. (2020)
3	ULK1(ATG1)	Initiates autophagy	Forms a complex with ATG13 and FIP200 to start phagophore formation.	Initiates autophagy upon activation by AMPK/mTOR	Lee et al. (2021a), Israeli et al. (2022)
4	ATG5-ATG12	Autophagosome membrane elongation	Forms a conjugation system with ATG16L1 to elongate the isolation membrane.	Promotes autophagosome formation	Romanov et al. (2012), Chen et al. (2014)
5	LC3(ATG8)	Maturation of autophagosomes	Conjugated to PE to form LC3-ll, which incorporates into autophagosome membranes.	Facilitates cargo sequestration and degradation	Chen et al. (2012), Bansal et al. (2018)
6	Noncoding RNA	Post-transcriptional regulation of autophagy	miRNA (e.g., miR-101) and lncRNAs modulate expression of key autophagy gene (e.g., ATG4D).	Modulates autophagy gene expression	Zhang et al. (2017); Ma et al. (2022b)

Abbreviations: mTOR: mechanistic target of rapamycin; AMPK: AMP-activated protein kinase; ULK1 (ATG1): Unc-51-like autophagy activating kinase 1 (Autophagy-related gene 1); ATG5: Autophagy-related protein 5; ATG12: Autophagy-related protein 12; LC3 (ATG8): Microtubule-associated protein 1A/1B-light chain 3 (Autophagy-related protein 8); PE: phosphatidylethanolamine; miRNA: MicroRNA; lncRNA: Long non-coding RNA; ATG4D: Autophagy-related gene 4D.

#### 3.3.1 ATG gene family (autophagy-related genes)

**FIGURE 4:**
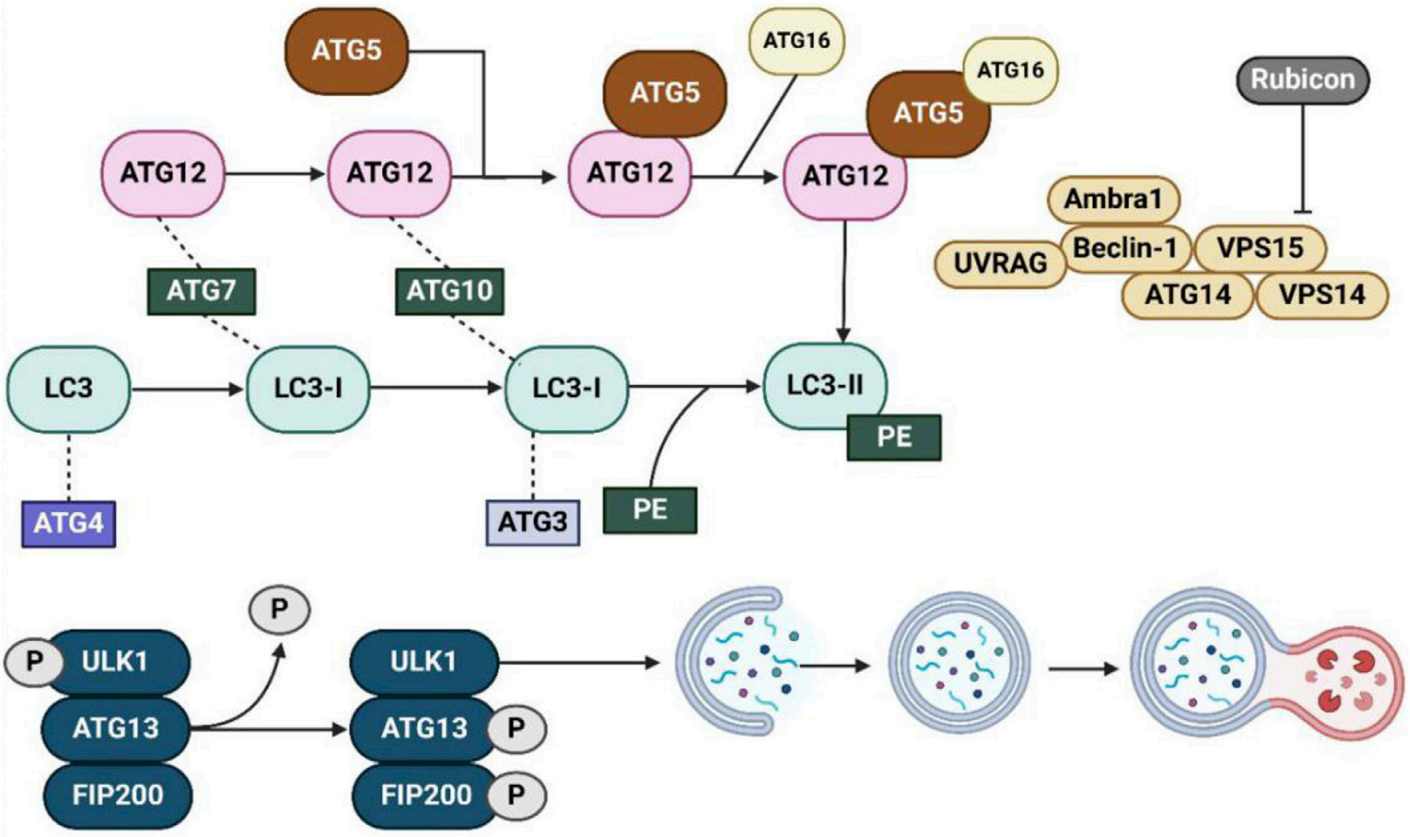
The autophagy pathway and key regulatory proteins involved in autophagosome formation and maturation. This diagram provides an overview of the molecular machinery involved in the formation and maturation of autophagosomes. The process begins with the activation of the ULK1 complex (composed of ULK1, ATG13, and FIP200), which is regulated by phosphorylation (indicated by P). This complex initiates the formation of the phagophore, which is extended and closed to form the autophagosome. Several key autophagy-related proteins (ATGs), such as ATG12, ATG5, ATG7, and ATG16, are involved in this process, facilitating the elongation of the autophagosomal membrane and the incorporation of LC3 (converted from LC3-I to LC3-II via conjugation with PE). Beclin-1, UVRAG, and other associated proteins like Ambra1, VPS15, ATG14, and VPS14 regulate the nucleation and expansion of the autophagosome. The pathway also includes the regulatory role of Rubicon, a key protein that inhibits the activation of the PI3K complex, thereby influencing the autophagic process. This network of interactions ensures the proper execution of autophagy, including the removal of damaged cellular components and the maintenance of cellular homeostasis. Abbreviations: ULK1: Unc-51-like autophagy activating kinase 1; ATG: Autophagy-related gene; LC3: Microtubule-associated protein 1A/1B-light chain 3; PE: Phosphatidylethanolamine; Rubicon: RUN domain and cysteine-rich domain-containing protein; Beclin-1: Autophagy-related protein 6; UVRAG: UV radiation resistance-associated gene; Ambra1: Autophagy and Beclin-1 regulator 1; VPS15: Vacuolar protein sorting 15; VPS14: Vacuolar protein sorting 14; FIP200: Focal adhesion kinase family interacting protein of 200 kDa. Reproduced from Liu, Beibei et al. Targeting cell death mechanisms: the potential of autophagy and ferroptosis in hepatocellular carcinoma therapy. Frontiers in immunology vol. 15 1450487, Copyright ^©^ 2024 by (Liu B. et al., 2024).

The ATG (Autophagy-related) gene family plays a crucial role in regulating the autophagic process, with each member contributing to distinct stages, from initiation to degradation (Li and Zhang, 2019). Here, we list some key members of the ATG gene family and their specific roles (Figure 4). ULK1, the human counterpart of yeast ATG1, is a component of the ULK1 complex, which also includes ATG13 and FIP200 (Mizushima, 2010; Tabata et al., 2024). This complex is essential for the induction of autophagy and is directly activated or inhibited by upstream signals like mTOR and AMPK (Chen et al., 2018a). Upon activation, ULK1 phosphorylates other downstream ATG proteins to trigger the formation of the phagophore (Zachari and Ganley, 2017). The ATG5-ATG12/ATG16L1 complex functions as a conjugation system, playing an essential role in the elongation of the autophagosome membrane (Hwang et al., 2012; Tan et al., 2020). Specifically, ATG12 covalently binds to ATG5, and together with ATG16L1, they form a complex that facilitates autophagosome formation (Hwang et al., 2012; Ji et al., 2019). This complex recruits LC3 (ATG8) to the membrane, enabling its expansion and closure around the cargo (Park et al., 2022; Varga et al., 2022). While LC3 (ATG8) is a critical protein involved in the maturation of the autophagosome (Jacquet et al., 2021; Gluschko et al., 2022). It exists in two forms: LC3-I (cytosolic) and LC3-II (membrane-bound) (Hu et al., 2014; Zou et al., 2015). During autophagy, LC3 is conjugated to phosphatidylethanolamine (PE) (Nakatogawa, 2013), which then converts LC3-I to LC3-II (Hu et al., 2014). LC3-II is then incorporated into the autophagosomal membrane. This modification marks the cargo and promotes autophagosome formation, as well as its fusion with lysosomes. ATG9, the only known transmembrane ATG protein, is involved in supplying membranes to the growing phagophore (Tamura et al., 2010). It cycles between different membrane compartments, supplying lipids for autophagosome formation (Choi et al., 2024).

#### 3.3.2 mTOR and AMPK signaling pathways

Autophagy is primarily regulated by the mTOR and AMPK signaling pathways, which respond to cellular energy levels and nutrient availability (Li Y.-Y. et al., 2024; Mundo Rivera et al., 2024). Typically, mTOR functions as a key inhibitor of autophagy by suppressing the ULK1 complex. Under nutrient-rich conditions, mTOR is active and phosphorylates ULK1, thereby preventing the initiation of autophagy (Nazio and Cecconi, 2017; Xu and Wan, 2023). This mechanism ensures that, when energy levels are high, the cell prioritizes growth and biosynthesis over degradation. Conversely, when mTOR activity is diminished, such as during starvation or oxidative stress, ULK1 undergoes dephosphorylation and activation, initiating autophagy to degrade cellular components and provide energy (D’Amico et al., 2022). While AMPK is activated in response to low cellular energy (high AMP/ATP ratio). When it is activated, AMPK promotes autophagy by inhibiting mTOR through direct phosphorylation of TSC2 and Raptor (negative regulators of mTOR) (Lee et al., 2010). Additionally, AMPK can directly activate ULK1, bypassing mTOR inhibition and stimulating autophagy (Egan et al., 2011; Wang S. et al., 2022). We believe this dual regulation ensures that autophagy is rapidly triggered in response to cellular energy stress, and providing an adaptive mechanism to restore metabolic balance. The interplay between mTOR and AMPK is a finely tuned system that ensures autophagy is only activated under conditions of stress, where catabolic processes like autophagy are necessary for survival. For example, in photoaging, excessive UV-induced oxidative stress can inhibit mTOR activity, thereby promoting autophagy to clear damaged proteins and organelles and protect the skin from further damage (Wang X. et al., 2023).

#### 3.3.3 Recent advances in autophagy regulation

Recent research has uncovered additional regulators of autophagy, expanding our understanding of this complex process (Chowdhury and Karmakar, 2023; Almujri and Almalki, 2024; Lin et al., 2024b). Several novel ATG proteins have been identified, enhancing the diversity of autophagy regulation. For example, ATG101, a relatively recent discovery, has been shown to stabilize the ULK1 complex and facilitate the early stages of autophagy (Gallagher and Chan, 2013). This protein plays a critical role in the proper formation of autophagic initiation complexes, particularly under stress conditions (Mizushima, 2010; Corona Velazquez and Jackson, 2018). Furthermore, emerging evidence indicates that non-coding RNAs (ncRNAs), including microRNAs (miRNAs) and long non-coding RNAs (lncRNAs), are significantly involved in autophagy regulation (Almujri and Almalki, 2024; Liu M. et al., 2024; Yu S. et al., 2024). Specifically, these ncRNAs regulate the expression of autophagy-related genes at the post-transcriptional level. For example, miR-101 has been shown to inhibit autophagy by directly targeting ATG4D, a key protein in the LC3 processing pathway (Fu et al., 2017). Likewise, certain lncRNAs, such as HOTAIR, have been implicated in either promoting or suppressing autophagy depending on the context (Li et al., 2021; Luo et al., 2024). These findings underscore the intricate regulatory network of autophagy, encompassing both traditional protein pathways and novel RNA-based mechanisms.

So far, these advances have indicated that autophagy is regulated by a complex interplay of protein-based pathways and non-coding RNA molecules (Kolapalli et al., 2023; Kumar et al., 2023), which opens new avenues for therapeutic interventions. For example, modulating autophagy through small molecules, gene therapy, or RNA-based therapies could offer potential treatments for conditions related to autophagy dysfunction, such as neurodegenerative diseases, cancer, and photoaging (Li T. et al., 2019). In the context of skin photoaging, strategies aimed at enhancing autophagic activity could help clear damaged cellular components, reduce oxidative stress, and improve skin regeneration (Suresh et al., 2020; Whitmarsh-Everiss and Laraia, 2021). Understanding these mechanisms is essential for identifying innovative strategies to modulate autophagy in disease contexts, including skin photoaging, where autophagy dysregulation may worsen aging-related cellular damage (Yu T. et al., 2015; Wang et al., 2019).

The regulation of autophagy involves an intricate network of signaling pathways, gene products, and emerging molecular regulators, such as non-coding RNAs. These pathways and molecules collaborate to preserve cellular homeostasis, especially in response to stress factors like those implicated in skin photoaging. Gaining a deeper understanding of these regulatory systems provides valuable insights into potential therapeutic targets for diseases associated with autophagy dysfunction.

## 4 Interaction between autophagy and photoaging

### 4.1 Protective role of autophagy in counteracting photoaging

Autophagy can facilitate the removal of damaged cellular components and maintains cellular homeostasis, and at the same time, it also plays a vital protective role in alleviating the effects of photoaging in this way (Hao et al., 2019; Ma J. et al., 2022). One of the primary functions of autophagy is the elimination of dysfunctional organelles and aggregated proteins, thereby reducing the production of ROS (Peng et al., 2024; Vikram et al., 2024). Specifically, autophagy targets damaged mitochondria through a selective process, which is known as mitophagy (Tran and Reddy, 2020; Shang et al., 2022). By degrading these impaired mitochondria, autophagy prevents the excessive ROS production that arises from mitochondrial dysfunction (Xu et al., 2024). This reduction in oxidative stress is particularly critical for protecting skin cells from the harmful effects of UV exposure, thereby contributing to skin health and enhancing resilience against aging (Bai et al., 2021).

Moreover, autophagy plays an integral role in maintaining DNA stability and promoting DNA repair mechanisms (Zhao et al., 2012). During photoaging, UV radiation induces various forms of DNA damage, which can lead to mutations that accelerate cellular aging (Al-Sadek and Yusuf, 2024; Markovitsi, 2024). However, autophagy aids in the removal of damaged proteins and organelles that may carry genetic defects, thereby minimizing the accumulation of harmful mutations (Vlada et al., 2015; Luo et al., 2023). Additionally, autophagy is associated with the expression of proteins involved in DNA repair pathways (Ye et al., 2017), ensuring efficient repair of UV-induced DNA damage. Therefore, by preserving genomic integrity and enhancing repair processes, autophagy significantly reduces the risk of genetic mutations associated with photoaging, reinforcing its critical role in skin protection and longevity (Vikram et al., 2024; Zhong et al., 2024).

### 4.2 Effects of UV radiation on autophagy

UV radiation has a dual effect on autophagy, acting both as an inducer and a disruptor of this essential cellular process. Specifically, UV exposure can trigger autophagy by activating pathways that inhibit mTOR or by stimulating AMPK.

#### 4.2.1 UV-induced autophagy activation

We find that UV radiation can inhibit mTOR, which is a key negative regulator of autophagy (Yang et al., 2013; Huangfu et al., 2023). To be brief, when mTOR activity is reduced, the autophagic machinery is activated, enabling cells to initiate the degradation of damaged organelles and proteins (Al-Bari and Xu, 2020). The inhibition of mTOR allows for the upregulation of ATGs, which are crucial for the formation of autophagosomes. This initiates the sequestration of damaged components such as proteins, lipids, and damaged mitochondria (Boutouja et al., 2019). Furthermore, UV radiation can activate AMPK, an energy sensor that plays a crucial role in responding to cellular energy depletion (Lim C. et al., 2024; Xia et al., 2024). AMPK activation further supports autophagy by increasing the catabolic processes within the cell, including the enhancement of lysosomal function and the promotion of autophagosome-lysosome fusion (Paquette et al., 2021; Xu and Wan, 2023). As cellular energy levels drop due to UV-induced stress, AMPK activation promotes autophagy as a protective mechanism (Zhao et al., 2016). By activating this dual pathway, cells efficiently clear damaged components, alleviate oxidative stress, and ultimately adapt to the challenges imposed by UV exposure (Cao and Wan, 2009; Lee et al., 2014).

#### 4.2.2 Autophagy dysfunction from excessive UV radiation

However, excessive UV radiation can overwhelm the cellular repair mechanisms, then leading to autophagy dysfunction finally (Wang B.-J. et al., 2024). Because too prolonged or high doses of UV exposure can cause a state of autophagic flux disruption (Wang B.-J. et al., 2024), which is characterized by impaired fusion with lysosomes, resulting in the accumulation of autophagosomes. This accumulation prevents the clearance of damaged cellular materials and instead leads to their buildup (Farizatto et al., 2017; Zhang et al., 2021). This disruption of autophagic flux is linked to the overproduction of ROS and the depletion of cellular ATP, which further inhibit the proper functioning of autophagic machinery (Yamamoto et al., 2021).

In extreme cases, excessive UV radiation can trigger autophagic cell death, where the autophagic process, initially serving a protective role, becomes a mechanism for cell demise (Hansda and Ghosh, 2022; Guerrero-Navarro et al., 2024). In this context, excessive autophagy leads to the degradation of essential cellular components, contributing to cell death and tissue damage, which worsens the overall effect of UV-induced photoaging (Xia et al., 2024). The dysregulation of autophagy in this scenario is associated with the overproduction of ROS, which not only overwhelms the cell’s antioxidant defenses but also interferes with the signaling pathways necessary for maintaining proper autophagic function (Wang D.-K. et al., 2022). This intricate interplay between autophagy activation and dysfunction emphasizes the delicate balance cells must maintain when responding to UV stress, highlighting the dual nature of autophagy as both a protective and potentially harmful process in the context of photoaging (Lamore and Wondrak, 2013).

### 4.3 Regulation of photoaging-related processes by autophagy

**FIGURE 5:**
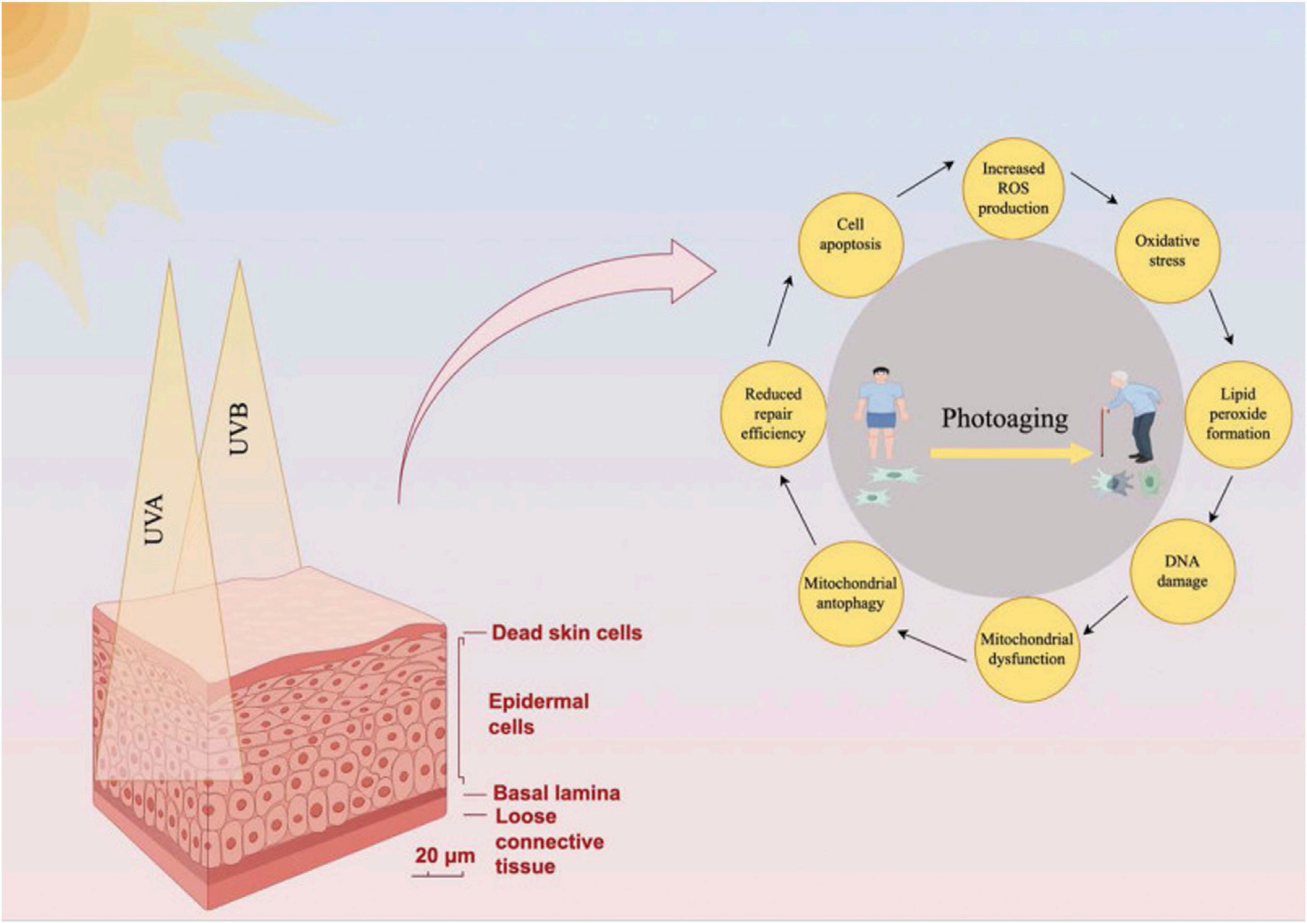
Regulation of photoaging-related processes by autophagy. This diagram illustrates how autophagy mitigates photoaging induced by UV radiation (UVA and UVB). The left side presents a cross-sectional view of skin (scale bar: 20 μm), showing epidermal cells, basal lamina, and loose connective tissue, with UV penetration causing dead skin cell accumulation. The right side features a circular flowchart centered on photoaging—depicted as a young individual aging into an elderly person—outlining key mechanisms: 1) mitochondrial dysfunction, 2) reduced repair efficiency, 3) cell apoptosis, 4) increased reactive oxygen species (ROS) production, 5) lipid peroxide formation, and 6) DNA damage. These processes are interconnected, with arrows indicating their cyclical nature. Autophagy regulates inflammation (e.g., via NF-κB suppression and NLRP3 inflammasome inhibition), cell cycle progression (e.g., halting the cycle for DNA repair), and apoptosis (balancing cell survival and death). Mitophagy, a subset of autophagy, counters mitochondrial dysfunction by removing damaged mitochondria, reducing ROS and oxidative stress. However, as autophagic function declines with age—due to reduced expression of genes like ATG7 and LC3—the skin’s ability to combat UV-induced damage diminishes, accelerating photoaging. Reproduced from Zhong et al. Role of autophagy in skin photoaging: A narrative review. Medicine, Copyright ^©^ 2022 by (Zhong et al., 2024).

Autophagy plays a multifaceted role in regulating several processes associated with photoaging, including inflammation, cell cycle regulation, and apoptosis (Chen et al., 2017; Yang Y. et al., 2024). Through these mechanisms, autophagy mitigates cellular damage and slows down skin aging caused by UV radiation (Figure 5). However, as autophagic function declines with age, the ability to combat these effects diminishes, which leads to accelerated photoaging (Martic et al., 2020).

#### 4.3.1 Regulation of inflammatory responses

Chronic low-grade inflammation, often termed inflammaging, is a hallmark of both chronological aging and photoaging (Salminen, 2022; Salminen et al., 2022). Autophagy plays a crucial role in regulating inflammation by modulating key inflammatory signaling pathways (Figure 5). One of the primary mechanisms involves the suppression of NF-κB signaling, which is a central pathway in the inflammatory response (Chaudhary et al., 2024; Xue et al., 2024). For instance, when UV radiation induces oxidative stress and DNA damage, NF-κB is activated (Chen B. et al., 2022), then leading to the production of pro-inflammatory cytokines such as IL-6 and TNF-α (Tomasello et al., 2022). Moreover, autophagy limits this inflammatory response by selectively degrading inflammatory mediators and damaged organelles, including dysfunctional mitochondria, which are significant sources of ROS (Liu H. et al., 2024).

Additionally, autophagy interacts with the NLRP3 inflammasome, a critical component responsible for producing IL-1β, a potent pro-inflammatory cytokine (Gupta et al., 2024), a potent pro-inflammatory cytokine (Gupta et al., 2024; Wang T. et al., 2024). By degrading damaged mitochondria and preventing the release of mitochondrial DNA and ROS, autophagy inhibits inflammasome activation (Cui et al., 2023), thus reducing chronic inflammation. This anti-inflammatory effect of autophagy helps protect skin cells from the persistent inflammation that accelerates photoaging (Liu et al., 2013).

#### 4.3.2 Cell cycle regulation and apoptosis

Autophagy exerts significant influence over the regulation of the cell cycle and apoptosis, playing a dual role in determining cell fate in response to UV-induced stress (Sample and He, 2017). On the one hand, autophagy helps to maintain proper cell cycle progression by degrading cyclins and other cell cycle regulators (Rambold and Lippincott-Schwartz, 2011; Mestre Citrinovitz et al., 2019). Specifically, in response to UV-induced DNA damage, autophagy temporarily halts the cell cycle to allow DNA repair (Lo et al., 2005; Wu et al., 2007), preventing the propagation of mutations that contribute to aging and carcinogenesis. This process not only preserves genomic stability but also serves as a protective mechanism to slow the photoaging process (Wang et al., 2021; Xie H. et al., 2022).

Autophagy and apoptosis are interconnected processes, with autophagy often acting as a survival mechanism to delay or prevent apoptosis under moderate stress conditions (Yeo et al., 2016; Saoudaoui et al., 2021). However, when stress becomes excessive, such as with prolonged UV exposure, autophagy may promote apoptotic cell death (Vikram et al., 2024). This dual role allows autophagy to balance cell survival and death, ensuring that severely damaged cells are removed while preserving those with repairable damage. Furthermore, chronic inflammation, known as inflammaging, can be triggered by excessive UV exposure and prolonged cellular stress (Lee Y. I. et al., 2021). Inflammaging refers to the persistent low-grade inflammation that occurs with aging and has been linked to various age-related diseases, including skin aging (Lee Y. I. et al., 2021; Pilkington et al., 2021). The interplay between autophagy, inflammation, and apoptosis is crucial in modulating skin aging, as sustained inflammation exacerbates cellular damage and accelerates the aging process. By regulating apoptosis, autophagy prevents the accumulation of dysfunctional cells, thereby mitigating skin aging (Song et al., 2017; Ho and Dreesen, 2021).

#### 4.3.3 Autophagy decline and accelerated photoaging

As age progresses, autophagic function gradually declines, reducing the cellular capacity to manage UV-induced damage (Wang et al., 2019). This decline in autophagy is primarily due to age-related alterations in the autophagic machinery, including decreased expression of autophagy-related genes and impaired autophagic flux (Chen et al., 2018a). These changes result in an inability to efficiently remove damaged proteins, organelles, and other cellular debris, which accumulate over time (Lim S. H. Y. et al., 2024). Studies have shown that this age-associated decline in autophagic activity contributes to the impaired response of skin cells to environmental stressors like UV radiation (Huang et al., 2019). Consequently, this decline in autophagy exacerbates photoaging by failing to prevent the buildup of damaged proteins, organelles, and ROS (Figure 5). For instance, mitophagy—the selective removal of damaged mitochondria—becomes less efficient, leading to increased mitochondrial dysfunction and oxidative stress (Di Rienzo et al., 2024; Kong et al., 2024). As a result, the skin becomes more vulnerable to UV-induced DNA damage, protein aggregation, and chronic inflammation, all of which accelerate the aging process (Chaudhary et al., 2023).

A notable example of age-related decline in autophagy is the reduced expression of autophagy-related genes, such as ATG7 and LC3, in older individuals (Yu P. et al., 2015). Lower levels of these crucial proteins impair the autophagic process, resulting in the accumulation of cellular debris and increased susceptibility to UV-induced damage (Baechler et al., 2019; Caution et al., 2019). This reduction in autophagic activity associated with aging not only accelerates photoaging but also increases the risk of skin disorders, including carcinogenesis. Understanding the role of autophagy in these processes emphasizes its potential as a therapeutic target to alleviate the effects of UV-induced skin aging.

In conclusion, autophagy plays a crucial role in regulating inflammatory responses, cell cycle progression, and apoptosis in the context of photoaging (Zhao et al., 2022; Xia et al., 2024). However, as autophagic function declines with age, the skin becomes more susceptible to UV-induced damage, inflammation, and premature aging.

## 5 Recent research progress

### 5.1 Findings from *in vitro* and *in vivo* studies

*In vitro* studies have significantly advanced our understanding of autophagy’s role in responding to UV exposure and its protective effects against photoaging (Prasanth et al., 2019; Martic et al., 2020). Research has shown that UV radiation stimulates autophagic activity in skin cells, primarily through mechanisms involving the inhibition of mTOR and activation of AMPK (Lee et al., 2019; Chu et al., 2023). This autophagic activity aids in the clearance of damaged mitochondria and proteins, thus contributing to cellular protection (Gao et al., 2023; Zamanian et al., 2024). Building on these findings, *in vivo* studies using animal models, such as mice and zebrafish, reinforce autophagy’s critical role in mitigating UV-induced skin damage (Li M. et al., 2024; Tang Q.-Q. et al., 2024). Mice with impaired autophagic function exhibit increased sensitivity to UV radiation, leading to more pronounced signs of photoaging (Wang et al., 2019; Wang et al., 2021). While models with enhanced autophagic activity show reduced skin damage and better preservation of skin integrity (Konger et al., 2016; de Medeiros et al., 2018; Khater et al., 2024).

### 5.2 Clinical studies and applications

The potential of autophagy modulators, such as rapamycin and metformin, has attracted considerable clinical interest for addressing photoaging (Zhang et al., 2016; Qin D. et al., 2018). Rapamycin, a well-known mTOR inhibitor, has shown promise in clinical trials by enhancing autophagic activity (Palma et al., 2022; Aitken et al., 2023; Redl et al., 2024). It facilitates the clearance of damaged cellular components, resulting in improvements in skin texture, wrinkle depth, and elasticity in older individuals (Rangwala et al., 2014). Similarly, metformin, an AMPK activator commonly used in the treatment of diabetes (Pozzi et al., 2024), has demonstrated the ability to reduce oxidative stress and promote DNA repair in UV-exposed skin (Chen Q. et al., 2022). However, the use of pharmaceuticals like rapamycin and metformin can also present potential side effects, including immune suppression, metabolic disturbances, and gastrointestinal issues, which need to be carefully considered in long-term applications (Foretz et al., 2023). Beyond pharmaceuticals, the skincare industry has started incorporating autophagy-activating ingredients like resveratrol, curcumin, and spermidine into topical anti-aging products (Mundo Rivera et al., 2024). These compounds are known to stimulate autophagic processes within the skin, potentially aiding in the degradation of damaged proteins and organelles (Mostafa et al., 2021). Despite their potential, there are concerns about the long-term use of these compounds, as they may cause skin irritation or allergic reactions in some individuals. Moreover, the efficacy of these ingredients in stimulating autophagy through topical application is still debated, as their absorption and bioavailability may be limited by the skin barrier (Huang, 2020). While pharmaceutical agents like rapamycin and metformin exert systemic effects through oral ingestion, topical skincare ingredients are intended to work locally, directly affecting the skin where UV-induced damage occurs (Bai et al., 2021; Chen Q. et al., 2022). Theoretically, these ingredients might promote skin rejuvenation by enhancing the skin’s autophagic capacity, thereby addressing photoaging at a cellular level (Mostafa et al., 2021). However, the delivery of these compounds through the skin and their effectiveness in activating autophagy remain areas that need further research, as the bioavailability and penetration of topical agents differ significantly from oral pharmaceuticals (Stacchiotti and Corsetti, 2020; Chen M. et al., 2021). While initial studies report improvements in skin hydration, elasticity, and fine lines, further research is needed to validate the long-term effects of these agents on skin health and photoaging prevention (Thornfeldt and Rizer, 2016; Wang et al., 2024c; Xia et al., 2024).

### 5.3 Application of emerging technologies in research

Emerging technologies, such as single-cell RNA sequencing (scRNA-seq) and high-resolution imaging, have significantly advanced our understanding of autophagy and its role in photoaging (Pan et al., 2021; Lin et al., 2022). scRNA-seq enables researchers to analyze gene expression at the individual cell level (Popp et al., 2002; Bell et al., 2018), revealing changes in autophagy-related genes such as ATG5, LC3, and p62 in UV-exposed skin cells (Kim et al., 2018; Mahbubfam et al., 2022). This approach has identified distinct subpopulations of cells with varying levels of autophagic activity, helping to pinpoint which cells are more susceptible to UV-induced damage (Bernard et al., 2020). Complementing this, high-resolution imaging techniques, such as live-cell confocal microscopy, provide real-time visualization of autophagic structures and processes in UV-exposed cells (Chen R. et al., 2023; Maib et al., 2024), including mitochondrial dysfunction and protein aggregation (Beyer et al., 2024). Other innovative methods, like CRISPR/Cas9 gene editing, have been employed to investigate specific autophagy-related genes (Rahman et al., 2022), enhancing our understanding of their roles in protecting against UV damage (Huang et al., 2021). Furthermore, metabolomics analysis complements these technologies by profiling metabolic changes during autophagy activation in response to UV exposure, shedding light on the metabolic support autophagy provides for cellular repair in photoaging (Liang et al., 2020).

Next, Table 6 summarizes the clinical applications and emerging technologies related to autophagy modulation and their potential for mitigating photoaging, highlighting key agents and methodologies used in recent research.

**TABLE 6:** Summary of autophagy modulation in photoaging.

S. No.	Category	Technology/agent	Description	Key findings/applications	References
1	Clinical application	Rapamycin	mTOR inhibitor enhancing autophagic activity.	Improves skin texture, reduces wrinkles, and enhances elasticity inked to decreased collagen degradation.	Palma et al. (2022), Aitken et al. (2023), Redl et al. (2024)
		Metformin	AMPK activator with autophagy inducing properties.	Reduces oxidative stress and improves DNA repair in UV-exposed skin; potential for reducing photoaging markers.	Chen et al. (2022b), Pozzi et al. (2024)
2	Skincare products	Autophagy activating ingredients	Inducing properties. Includes compounds like resveratrol curcumin, and spermidine.	Claims to rejuvenate skin by promoting degradation of damaged components; moderate improvements in hydration and elasticity reported spermidine.	Thornfeldt and Rizer (2016), Stacchiotti and Corsetti (2020), Wang et al. (2024c)
3	Emerging technologies in research	Single-Cell RNA sequencing (scRNA-seq)	Analyzes individual cell gene expression.	Identifies changes in autophagy-related gene expression (e.g., ATG5, LC3, p62) in UV-exposed cells; reveals cellular subpopulations with varying autophagic activity.	Kim et al. (2018), Mahbubfam et al. (2022)
		High-resolution imaging	Real-time visualization of autophagic processes.	Tracks autophagic structures in UV-exposed cells; studies structural damage and protective role of autophagy against UV-induced stress.	Indira et al. (2018), Pellacani and Argenziano (2022), Chen et al. (2023a), Maib et al. (2024)
		CRISPR/Cas9 gene editing	Modifies specific autophagy-related genes.	Investigates the roles of genes (e.g., ATG7, BECN1) in preventing UV-induced damage and maintaining skin integrity.	Ha et al. (2017), Huang et al. (2021), Rahman et al. (2022)
		Metabolomics analysis	Profiles metabolites during autophagy.	Explores metabolic shifts during autophagy activation in response to UV exposure; elucidate autophagy’s role in energy homeostasis.	Liang et al. (2020), Chen et al. (2021b)

## 6 Conclusion

Autophagy is essential for protecting against photoaging by eliminating damaged organelles, breaking down aggregated proteins, and sustaining cellular homeostasis under UV-induced stress. The molecular processes underlying autophagy’s protective action include the removal of ROS-damaged mitochondria, regulation of inflammatory pathways, and facilitation of DNA repair. The modulation of autophagy via pathways such as mTOR and AMPK, alongside emerging regulators like non-coding RNAs, underscores its critical function in mitigating skin damage caused by UV radiation. Despite its promising therapeutic potential for counteracting photoaging, several challenges remain. These include the difficulty of monitoring autophagy dynamically in real-time, particularly *in vivo*, and the complex, context-dependent role of autophagy in photoaging. Additionally, the long-term safety and efficacy of autophagy modulators, as well as the consistency of benefits across diverse populations, require further evaluation.

In summary, while significant progress has been made in understanding autophagy’s role in photoaging, addressing these challenges through continued research will be essential to realizing its full therapeutic potential.

## 7 Future direction

To overcome the challenges in autophagy research related to photoaging, a comprehensive research strategy is necessary. Mechanistic studies should further explore the molecular processes by which autophagy regulates UV-induced DNA repair, inflammation, and mitochondrial function under oxidative stress. Investigating how autophagy interacts with other pathways involved in photoaging, such as apoptosis and cellular senescence, could enhance our understanding of its broader role in skin aging (Mostafa et al., 2022; Zhao et al., 2022; Han et al., 2024; Xia et al., 2024). The identification of novel autophagy regulators presents an exciting opportunity for advancement. Beyond well-established regulators like mTOR and AMPK, less-explored pathways involving non-coding RNAs and post-translational modifications could offer new approaches to fine-tune autophagic responses to UV damage (Türei et al., 2015; Li et al., 2020). Discovering molecules that modulate autophagy with minimal side effects could pave the way for targeted anti-photoaging therapies (Bu and Singh, 2021; Qu and Lin, 2021). Collaborative, multidisciplinary efforts will be critical to advancing this field. Integrating cellular biology, molecular genomics, and clinical medicine could translate mechanistic insights into practical therapeutic strategies. The use of advanced tools such as CRISPR/Cas9, combined with cutting-edge imaging and metabolomics, could provide deeper insights into autophagy’s role in skin aging. Furthermore, personalized clinical trials based on individual autophagy profiles could optimize therapeutic approaches for preventing and treating photoaging (Kaeberlein, 2017; Schmidt et al., 2021; Guan et al., 2024). Both basic and clinical research will play pivotal roles in harnessing autophagy regulation to prevent photoaging and promote healthier skin aging (Voegeli et al., 2017; Vikram et al., 2024).

## Glossary

UV: Ultraviolet
UVB: Ultraviolet B
UVA: Ultraviolet A
ROS: Reactive Oxygen Species
AMPK: AMP-Activated Protein Kinase
mTOR: Mechanistic Target of Rapamycin
CPD: Cyclobutane Pyrimidine Dimer
MMP: Matrix Metalloproteinase
NF-κB: Nuclear Factor kappa-light-chain-enhancer of activated B cells
ERK: Extracellular Signal-Regulated Kinase
JNK: c-Jun N-terminal Kinase
ATG: Autophagy-Related Gene
LC3: Microtubule-Associated Protein 1A/1B-Light Chain 3
ULK1: Unc-51-like Autophagy Activating Kinase 1
FIP200: Focal Adhesion Kinase Family Interacting Protein of 200 kDa
Beclin-1: Autophagy-Related Protein 6
UVRAG: UV Radiation Resistance-Associated Gene
PINK1: PTEN-Induced Kinase 1
Parkin: E3 Ubiquitin-Protein Ligase
PI3K: Phosphoinositide 3-Kinase
ATG5: Autophagy-Related Protein 5
ATG7: Autophagy-Related Protein 7
ATG3: Autophagy-Related Protein 3
PE: Phosphatidylethanolamine
CMA: Chaperone-Mediated Autophagy
miRNA: MicroRNA
lncRNA: Long Non-Coding RNA
ATG8: Autophagy-Related Protein 8
mTORC2: Mechanistic Target of Rapamycin Complex 2
SRBD1: SREB Binding Domain 1
ATG4D: Autophagy-Related Gene 4D.
